# *Acinetobacter baumannii* Utilizes a Type VI Secretion System for Bacterial Competition

**DOI:** 10.1371/journal.pone.0059388

**Published:** 2013-03-19

**Authors:** Michael D. Carruthers, Paul A. Nicholson, Erin N. Tracy, Robert S. Munson

**Affiliations:** Center for Microbial Pathogenesis, The Research Institute at Nationwide Children’s Hospital and Department of Pediatrics, College of Medicine, The Center for Microbial Interface Biology, The Ohio State University, Columbus, Ohio, United States of America; Centre National de la Recherche Scientifique, Aix-Marseille Université, France

## Abstract

Type VI secretion systems (T6SS) are a class of macromolecular secretion machines that are utilized by a number of bacteria for inter-bacterial competition or to elicit responses in eukaryotic cells. *Acinetobacter baumannii* is an opportunistic pathogen that causes severe infections in humans. These infections, including pneumonia and bacteremia, are important, as they are often associated with hospitals and medical-settings where they disproportionally affect critically ill patients like those residing in intensive care units. While it is known that *A. baumannii* genomes carry genes whose predicted products have homology with T6SS-associated gene products from other bacteria, and secretion of a major T6SS structural protein Hcp has been demonstrated, no additional work on an *A. baumannii* T6SS has been reported. Herein, we demonstrated that *A. baumannii* strain M2 secretes Hcp and this secretion was dependent upon TssB, an ortholog of a bacteriophage contractile sheath protein, confirming that strain M2 produces a functional T6SS. Additionally, we demonstrated that the ability of strain M2 to out-compete *Escherichia coli* was reliant upon the products of *tssB* and *hcp.* Collectively, our data have provided the first evidence demonstrating function in inter-bacterial competition, for a T6SS produced by *A. baumannii.*

## Introduction

*Acinetobacter baumannii* are Gram-negative opportunistic human pathogens that have emerged as an important cause of severe nosocomial infections, which are becoming increasingly difficult to treat due to antibiotic resistance [1], [2]. While many of the members of the genus *Acinetobacter* are commonly found in soil, aquatic environments and on human skin, the specific natural reservoir(s) for *A. baumannii* remain(s) to be defined [1]. Infections caused by *A. baumannii* such as ventilator-associated pneumonia and surgical site infections generally occur in immune compromised patients, often with adverse outcomes [3]. To date, there are a limited number of reports that focus on the mechanisms by which *A. baumannii* colonizes surfaces and causes disease [1], [4], [5]. The reports include identification of a phospholipase D produced by *A. baumannii* that plays a role in serum resistance [6]. *Acinetobacter baumannii* ATCC19606^T^ relies upon an acinetobactin-mediated iron acquisition system to persist in the host and cause apoptosis in epithelial cells [7]. The *A. baumannii* K1 capsule confers significantly improved growth in human ascites fluid, human serum resistance and survival in a rat soft-tissue infection model [8]. Outer membrane protein A has been shown to play a role in biofilm formation [9], induce toxicity in eukaryotic cells [10] and mediate adherence to epithelial cells [9], [11]. The *A. baumannii* biofilm associated protein been shown to play a role in biofilm formation and is thought to play a role in *A. baumannii* association with human cells *in vitro* [12]. *Acinetobacter baumannii* 19606^T^ produces Csu pili, which mediate adherence to and biofilm formation on abiotic surfaces [13]. Despite these advances, we have a limited understanding of *A. baumannii* biology both in and around the human host.

Type VI secretion systems (T6SS) are a class of macromolecular secretion machines, analogous to the tail assembly of contractile bacteriophages [14], [15], which can facilitate the injection of effector proteins into target cells. Injection of effectors via T6SS into eukaryotic cells has been linked to phenotypes such as inhibition of phagocytosis [16] and induction of apoptosis [17], which could potentially explain the link between T6SS in certain bacteria and virulence [18]–[20].

T6SS also have a role in competition between bacterial species through the contact dependent injection of effectors, which may have bacteriostatic or bactericidal activities, in target bacteria [21]–[26]. Mougous and colleagues have identified [21] and characterized [24] 3 toxic T6SS secreted effectors (Tse1, Tse2, Tse3) produced by *Pseudomonas aeruginosa*. Tse2 is toxic to both bacteria and eukaryotic cells through a yet to be determined mechanism when expressed in the absence of its cognate immunity protein Tsi2. Tse1 and Tse3 have been shown to be toxic to bacteria. Russell *et al*. demonstrated that the most logical scenario for Tse1 and Tse3 toxicity is that these proteins are injected into a target bacterium’s periplasm, via T6SS, where Tse1 and Tse3 can then degrade peptidoglycan resulting in a comprised sacculus, which leads to cell lysis [24].

T6SS are comprised of at least 13 core proteins, which are widely conserved across known T6SS, and required for both T6SS biogenesis and function [27], [28]. The most notable of these proteins, due to its abundance in culture supernatants of T6SS expressing bacteria, is Hcp [29]. Hcp and orthologs are related to the major phage tail protein, gpV [15]. *In vitro*, Hcp can assemble into hexameric units that can subsequently assemble together [30]–[32]. Thus, it is possible that Hcp forms the filamentous tube-like structures that have been observed in association with the T6SS apparatus, similar to the T4 bacteriophage tail filament, which may facilitate passage of effectors from the initiating cell to the target cell [33]. Another core T6SS protein, TssB (VipA) is thought to be functionally analogous to a subunit of the T4 bacteriophage contractile sheath that, in complex with TssC and additional proteins, contracts to generate the mechanical force necessary to puncture biological membranes with the Hcp tube [29], [34].

It has recently been appreciated that the genomes of *Acinetobacter* spp. carry genes, which may encode components of T6SS [35], [36]. Hcp has been isolated and identified from *A. baumannii* 19606^T^ culture supernatants, indicating that the T6SS in this strain may be functional [36]. A role of for this *A. baumannii* T6SS has not been reported.

In this work, we identified a cluster of 20 genes and 3 additional genes carried on the chromosome of *A. baumannii* strain M2 that, together, are predicted to encode a T6SS. We observed that Hcp was a major protein component of strain M2 culture supernatants and secretion of Hcp was reliant upon the gene that is predicted to encode a T6SS sheath protein, TssB. These results indicated that strain M2 produced a functional T6SS. Further, we demonstrated that *A. baumannii* out-competes *Escherichia coli* in a T6SS- and contact-dependent manner. Overall, the data presented herein provide evidence for expression of a T6SS by *A. baumannii* M2 and demonstrate a role for this system in bacterial competition.

## Materials and Methods

### Bacterial Strains and Culture Conditions

All strains and plasmids are listed in Table 1. *Acinetobacter baumannii* strain M2, a clinical isolate from Cleveland Metro-Health Systems obtained from Philip Rather at Emory University, was used in this study [37]. All bacterial strains were grown on L-agar or L-broth prior to experimentation. Antibiotics were added to *A. baumannii* cultures, where appropriate, at the following concentrations in µg/ml: kanamycin, 20; chloramphenicol, 12.5; or ampicillin, 750. *Escherichia coli* cultures were supplemented with antibiotics at the following concentrations in µg/ml: kanamycin, 20; streptomycin, 25 or ampicillin, 50.

**Table 1:** Plasmids and bacterial strains included in the study.

Plasmid or strain	Relevant characteristic(s)	Reference/Source
**Plasmids**		
pFLP2	Carries the FLP recombinase gene	[42]
pRSM574	pBR322 with Tn903 kanamycin resistance cassette	This Study
pRSM2724	pRSM574 with *sacB* from pFLP2 cloned as an EcoRI fragment	This Study
pKD13	Contains the kanamycin resistance gene from Tn5 flanked by FRT sites and the R6K γ-*ori*	[48]
pGEM-T-Ez	General cloning plasmid	Promega
pRSM3575	pGEM containing *hcp* and flanking sequence	This study
pRSM3576	pRSM3575 after introduction of the Δ*hcp*::*kan-sacB* mutation	This study
pRSM3577	pGEM containing *tssB* and flanking sequence	This study
pRSM3578	pRSM3577 after introduction of the Δ*tssB*::*kan-sacB* mutation	This study
pSMART LC-Kan	General cloning plasmid	Lucigen
pRSM3542	pKD13 containing the *kan-sacB* cassette from pRSM2724 in place of the kanamycinresistance gene from Tn5	This study
pUC18T-Tn*7*-Gm	Carries mini-Tn*7*	[53]
pKNOCK-Km	Suicide plasmid with R6K γ-*ori*	[54]
pRSM3508	pSMART LC-Kan containing Tn*7*-Gm from pUC18-T-Gm	This study
pRSM3509	pRSM3508 with allelic replacement of *aacC1* with *bla* from pGEM-T-Ez	This study
pRSM3506	pKNOCK-Km after removal of the MCS	This study
pRSM3510	Mini-Tn*7*-Ap cloned into pRSM3506	This study
pRSM3594	pRSM3510 containing *tssB* with expression driven from the predicted *asaA*promoter in the mini-Tn*7*-Ap element	This study
**Strains**		
* Acinetobacter baumannii strain* M2	Metro Health Systems Clinical Isolate	[37]
M2Δ*hcp:*:*kan*-*sacB*	Strain M2 mutant with *hcp* replaced with *kan-sacB*	This study
M2Δ*hcp*	Strain M2 mutant containing a unmarked in-frame deletion of *hcp*	This study
M2Δ*tssB*::*kan*-*sacB*	Strain M2 mutant with *tssB* replaced with *kan-sacB*	This study
M2Δ*tssB*	Strain M2 mutant containing a unmarked in-frame deletion of *tssB*	This study
M2Δ*tssB* Comp.	M2Δ*tssB* with mini-Tn*7*-Ap carrying *P_asaA_*-*tssB* incorporated in the *att*Tn*7* sitedownstream of *glmS2*	This study
* Escherichia coli* DH10B	General cloning strain, competitor	Invitrogen
* E. coli* DH5α	General cloning strain	Invitrogen
* E. coli* EC100D	General cloning strain, *pir* ^+^	Epicentre
* E. coli* DY380	Recombineering strain	[55]
* E. coli* DH5α(pFLP2)	Suicide plasmid that carries the FLP recombinase gene	[42]
* E. coli* HB101(pRK2013)	Helper strain for conjugation	[56]
* E. coli* EC100D(pTNS2*)*	Carries transposase gene for mini-Tn*7* transposition	[53]

### Bioinformatic Screen for T6SS Genes in *A. baumannii* Strain M2

The incomplete genome of *A. baumannii* strain M2 (Munson and Rather, unpublished) was screened for genes and/or proteins that showed sequence identity to genes or proteins associated with T6SS. T6SS genes and gene clusters were identified based on protein identity (NCBI BLAST, [38]), presence of conserved domains (NCBI Conserved Domains Search, [39]), and PHYRE2 [40] structural predictions compared to T6SS proteins in several bacteria.

### Recombinant DNA Methodologies

Primers, obtained from Integrated DNA Technologies (Coralville, IA), are listed in Table S1. We constructed unmarked in-frame deletions of *tssB* and *hcp* in the *A. baumannii* strain M2 chromosome essentially as was done for the construction of nontypeable *Haemophilus influenzae* mutants by Tracy *et al* [41]. This method uses the bacteriophage λ recombinase system to delete the gene of interest, replacing it with an FRT-*kan*-*sacB-*FRT cassette in a plasmid containing the gene of interest as well as flanking chromosomal DNA. This construct was used as a template for PCR and the amplicon used to transform M2. Subsequently, the cassette is removed by introduction of a suicide plasmid expressing the FLP recombinase into the mutant, which catalyzes a site-specific recombination event resulting in loss of the cassette. To construct strain M2 with an un-marked in-frame deletion of *hcp,* we amplified the strain M2 *hcp* gene and flanking sequence with primer set 4, then cloned the amplicon into pGEM-T-Easy (Promega, Madison, WI). An amplicon was also generated from pRSM3542 (construction described in Text S1) using primer set 3. This amplicon contained the FRT-*kan-sacB*-FRT region flanked on one side by 47 bp 5′ of *hcp* as well as the translational ATG start and on the other side by the last 21 bp of *hcp* and 29 bp downstream *of hcp*. The amplicon and the plasmid containing *hcp* were electroporated into *E. coli* DY380, which had been heat shocked at 42°C for 15 min prior to electroporation to induce expression of the λ recombinase system, then clones were selected on kanamycin-containing L-agar plates. Plasmids were isolated and characterized; a plasmid containing the FRT-*kan-sacB*-FRT cassette in place of *hcp* was saved as pGEM-Δ*hcp*::*kan-sacB*. An amplicon was generated with primer set 4 and pGEM-Δ*hcp*::*kan-sacB* as template, and the mutation was introduced into the M2 genome by natural transformation. Transformation in strain M2 was performed by incubating 1 µg of the amplicon with 500 µl of a strain M2 culture grown 2 h from a 1∶10 dilution of an overnight culture. This mixture was transferred to a L-agar plate and incubated at 37°C for 4 h. Cells were scraped from the plate into 500 µl of L-broth and dilutions were plated on L-agar containing 20 µg/ml of kanamycin. Genomic DNA was isolated from kanamycin resistant colonies, amplicons were prepared using primer set 4 and the allele exchange was confirmed by sequencing. A single clone was saved as M2Δ*hcp*::*kan*-*sacB*. pFLP2 [42], a suicide plasmid carrying the FLP recombinase was mated into M2Δ*hcp*::*kan-sacB*. Briefly, matings were performed by mixing 100 µl of each of stationary cultures normalized to an OD_600_ of 2.0 of the recipient strain (M2Δ*hcp*::*kan-sacB*), the helper strain *E. coli* HB101(pRK2013), *E. coli* DH5α (pFLP2) to 700 µl of warm L-broth pre-incubated at 32°C. This mixture was washed twice in 32°C L-broth followed by centrifugation at 7,000×*g*. After the final wash, the pellet was resuspended in 25 µl of 32°C L-broth and this suspension was spotted onto a pre-warmed L-agar plate and incubated overnight at 32°C. Plates were then moved to a 37°C incubator for 2 h to induce the FLP recombinase. Post-incubation, bacteria were swabbed from the agar surface and resuspended in 1 ml L-broth, vortexed on high for 8 sec and serial dilutions were plated on L-agar containing 12.5 µg of chloramphenicol and 10% sucrose. Plates were incubated at room temperature overnight. Strain M2 is resistant to chloramphenicol and was thus used to select against the *E. coli* donor in the mating mixture. The *sacB* gene product confers sucrose sensitivity to strain M2 growing at room temperature. Sucrose resistant and kanamycin sensitive clones were identified then analyzed by PCR and sequencing to confirm that the cassette was excised, resulting in an unmarked in-frame deletion. A single clone with the correct sequence was saved as M2Δ*hcp.* This procedure was followed to construct strain M2Δ*tssB* with an unmarked mutation in *tssB,* using primer sets 5 and 6 to make pGEM-*tssB* and pGEM-Δ*tssB*::*kan-sacB* respectively.

Complementation was performed using a mini-Tn*7* transposon system. A suicide vector that carries mini-Tn*7*-Ap, pRSM3510, was constructed as described in the Text S1. pRSM3510 constructs were introduced into *A. baumannii* via a four parental conjugal mating strategy modified from Kumar *et al*. [43]. Briefly, 100 µl of stationary cultures normalized to an OD_600_ of 2.0 of the recipient strain (*A. baumannii* mutants), *E. coli* HB101(pRK2013), *E. coli* EC100D(pTNS2) and *E. coli* EC100D(pRSM3510) were added to 600 µl of L-broth pre-incubated at 37°C. This mixture was washed twice in warm L-broth followed by centrifugation at 7,000×*g*. After the final wash, the pellet was resuspended in 25 µl of 37°C L-broth and this suspension was spotted onto a 37°C L-agar plate and incubated overnight at 37°C. Bacteria were then swabbed from the agar surface and resuspended in 1 ml L-broth, vortexed on high for 8 sec and serial dilutions were plated on L-agar containing ampicillin (750 µg/ml) and chloramphenicol (12.5 µg/ml) to select for *A. baumannii* cells containing mini-Tn*7-*Ap constructs integrated into the chromosome. To verify insertion downstream of *glmS2*, primer set 9 was used in PCR to amplify a 400 bp product that spans both downstream of *glmS2* and a portion of mini-Tn*7*-Ap. A clone with the correct sequence was saved as M2Δ*tssB*(*tssB^+^*).

### Growth Curve and Assay for Hcp Secretion

*Acinetobacter baumannii* strains were inoculated into 25 ml of L-broth in a 250 ml Erlenmeyer flask at an A_600_ of 0.05 and incubated at 37°C, 180 rpm. For growth curves, A_600_ was measured hourly for 6 h. To assay for Hcp secretion, cultures were grown until stationary phase (A_600_ ∼2.5) was reached. Cleared supernatants were obtained by centrifugation of cultures at 4,000×*g*, 4°C for 20 min followed by centrifugation of the resultant supernatant at 100,000×*g*, 4°C for 2 h. Twenty milliliters of cleared supernatants were concentrated 20-fold by filtration through Ultracel 10K centrifuge filters (EMD Millipore, Billerica, MA) according to the manufacturers protocol. The recovered concentrated cell supernatants were analyzed by SDS-PAGE as described below. Mass spectrometry was performed by The Ohio State University Mass Spectrometry and Proteomics Facility.

### SDS-PAGE and Western Blot Analysis

Protein separation was carried out by sodium dodecyl sulfate polyacrylamide gel electrophoresis (SDS-PAGE) as previously described [44]. Briefly, samples were mixed with an equal volume of 2× Laemmli sample buffer (Bio-Rad), boiled for 10 min, and cooled to room temperature. Samples were resolved on 4–20% Mini-Protean TGX Precast gels (Bio-Rad). Where needed, proteins were transferred from SDS-PAGE gels to nitrocellulose membranes as described by Tobwin *et al.* [45]. Membranes were blocked for 1 h with SuperBlock T20 (Thermo Fisher Scientific, Rockford, IL). A mouse α-Hcp6 antibody [46] at a dilution of 1/15,000 in Superblock T20 was used as a primary antibody for detection of Hcp. A horseradish peroxidase conjugated goat α-mouse secondary antibody (Thermo Fisher Scientific) at a dilution of 1∶5000 in Superblock was used followed by visualization of Hcp using the SuperSignal West Pico Chemiluminescent Substrate (Thermo Fisher Scientific) according to manufacturers instructions. Between application of secondary antibody and detection, nitrocellulose membranes were washed 3×5 min with tris-buffered saline with 0.05% Tween-20.

### Bacterial Competition Assays

Overnight L-broth cultures of bacterial strains were used to inoculate 25 ml of L-broth in 250 ml Erlenmeyer flasks to an A_600_ of 0.05. Cultures were incubated at 37°C, 180 rpm till an A_600_ of ∼0.5–0.6 was reached. Cultures were then diluted to an A_600_ of 0.4. For competition, 40 µl of *A. baumannii* was mixed with 4 µl of the competitor, *E. coli,* and 20 µl of this mixture was spotted on a pre-warmed agar L-agar plate. Plates were incubated at 37°C for 4 hr. Post-incubation, spots were excised from the plate, placed in a 14 ml culture tube containing 500 µl of PBS, vortexed for 5 s and serial diluted. Dilutions were plated on L-agar containing chloramphenicol to select for *A. baumannii* strains and on L-agar containing streptomycin to select for *E. coli* DH10B. In competition experiments with *E. coli* and *A. baumannii* strains separated by a filter, 20 µl of one strain was spotted on the agar surface, a 0.22 µm pore isopore filter (EMD Millipore) was placed on this spot and 20 µl of the other strain was spotted on top of the filter.

### Statistical Analysis

Statistical analyses were performed using non-parametric one-way analysis of variance (Kruskall-Walis). Post-hoc analysis was performed using Dunn’s multiple comparison test and a *P*-value <0.05 was considered to be statistically significant. Analyses were performed using GraphPad Prism version 6.0a (La Jolla, CA).

## Results

### Identification of a T6SS in *A. baumannii* Strain M2

**Figure 1:**
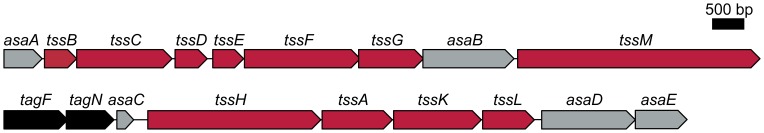
The predicted T6SS gene cluster of *A. baumannii* strain M2. A single ∼23 kb gene cluster, *asaA-tssBC-hcp-tssEFG-asaB-tssM-tagFN-asaC-tssHAKL-asaDE* carries 18 putative genes predicted to encode for components of a T6SS. The genome of *A. baumannii* strain M2 carries genes that are predicted to encode 12 of the 13 core T6SS proteins (*tss,* colored scarlet*)*, 2 proteins with homology to T6SS-associated proteins in other bacteria (*tag*, colored black*)* and 5 proteins encoded by genes only identified in *A. baumannii* T6SS gene clusters (*asa*, *Acinetobacter* type six secretion system-associated, colored gray). Three additional genes whose products have homology to VgrG were identified in other regions of the genome (data not shown).

While there is a proposed nomenclature for the genes encoding T6SS produced by *Acinetobacter* (*ass*) [36], in this manuscript we use the recently adopted standard T6SS nomenclature (*tss*) [47]. A draft sequence of the of *A. baumannii* strain M2 genome was generated (data not shown) and the resultant contig set was screened for genes and/or proteins that showed sequence homology to genes or proteins associated with T6SS. This targeted search resulted in the identification of a gene cluster of 18 open reading frames. Together with 3 additional genes outside of the cluster, we identified genes that are predicted to encode 12 of the 13 core T6SS proteins (Tss, Hcp and VgrG), 2 proteins associated with T6SS in other bacteria (Tag) and 5 proteins that appear to be encoded only in *Acinetobacter* spp. (Asa) (Genbank Accession KC432605-KC432608). A protein with identity to the core T6SS protein TssJ was not detected in the strain M2 predicted proteome. The genetic organization of the *A. baumannii* T6SS gene cluster, depicted in Figure 1, appears to be conserved amongst sequenced *A. baumannii* strains and is similar to the of T6SS gene cluster of *A. baylyi* ADP1 with the exception that a portion of the gene cluster is inverted (data not shown). The *tssB* and *hcp* genes, which are predicted to encode a putative T6SS sheath protein and the T6SS tube protein respectively, were chosen for mutational analysis.

### Construction and Characterization of Unmarked In-frame Insertion/deletion Mutants Lacking the *tssB* and *hcp* Genes in Strain M2

We used a modification of methods developed for use in *E. coli* by Wanner and co-workers [48] that employed both the bacteriophage λ recombination system as well as the yeast FLP recombinase system. In *E. coli*, the bacteriophage λ recombinase will catalyze site-specific recombination with 50 bp of homologous DNA. The FLP recombinase was used to delete DNA flanked by 2 FLP recombinase recognition sites (FRT). A mutant was constructed which contains sequences 5′ of the deleted gene, followed by short ORF containing the ATG start of the deleted gene, the single remaining FRT site and the last 21 nt of the deleted gene, followed by sequences 3′ to the gene. The 3′ portion of the deleted gene is retained to preserve the Shine-Dalgarno sequence 5′ of the downstream gene. Mutants with unmarked, in-frame deletions in the *tssB* and *hcp* genes were constructed and the deletions confirmed by sequencing.

### *Acinetobacter baumannii* Strain M2 Secreted Hcp

**Figure 2:**
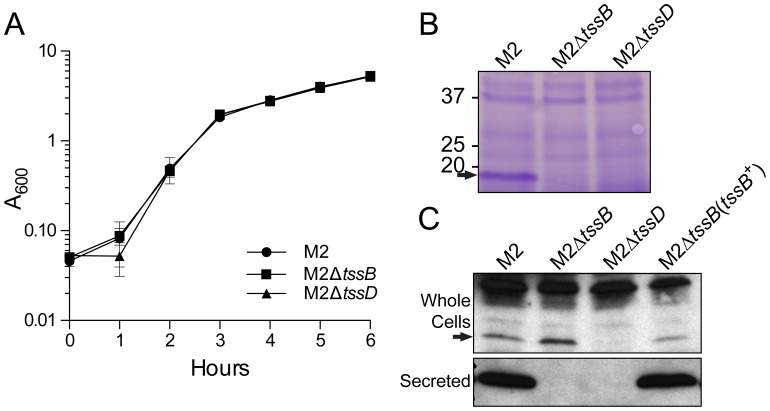
*Acinetobacter baumannii* strain M2 secreted Hcp. A. Growth curves of strain M2 and the isogenic *tssB* and *hcp* mutants. Three biological replicates were performed; error bars denote the standard error of the mean of the biological replicates. B. Concentrated culture supernatants from strain M2, M2Δ*tssB* and M2Δ*hcp* analyzed by SDS-PAGE and Coomassie staining. A band corresponding to the approximate molecular mass of Hcp (∼19 kDa), indicated by the arrow, was observed in the sample derived from strain M2 and was absent in the samples from M2Δ*tssB* and M2Δ*hcp*. Mass spectrometry confirmed that the major protein in this band was Hcp. C. Western blot analysis of whole cell lysates and concentrated culture supernatants of *A. baumannii* strains. Hcp, indicated by the arrow, was detected in whole cell lysates of strain M2, M2Δ*tssB* and the complemented *tssB* mutant as well as in concentrated culture supernatants of strain M2 and the complemented *tssB* mutant.

A hallmark of a functional T6SS is the secretion of Hcp [49]. To first determine if *A. baumannii* strain M2 produced a functional T6SS, we assessed the ability of the *tssB* and *hcp* mutants to secrete Hcp. The growth curves of strains M2, M2Δ*tssB* and M2Δ*hcp* were similar (Figure 2.A) indicating that *tssB* and *hcp* mutations did not significantly alter growth kinetics. To assay for Hcp secretion concentrated cell supernatants were prepared from stationary phase cultures of strain M2, the isogenic *tssB* and *hcp* mutants and the *tssB* complemented *tssB* mutant. The predominant band in concentrated cultures supernatants prepared from strain M2, as detected by SDS-PAGE and Coomassie staining, had the approximate molecular mass of Hcp (Figure 2.B) and was identified as Hcp by mass spectroscopy. In addition, the band was absent in samples prepared from cultures strains of M2Δ*tssB* and M2Δ*hcp*. An antibody that was generated against Hcp6 from *Burkholderia pseudomallei* [46] was used for Western blot analysis to determine the presence Hcp in whole cells and concentrated culture supernatants. Hcp was detected in whole cells of strain M2, M2Δ*tssB* and the complemented *tssB* mutant, but not in M2Δ*hcp*. In addition, Hcp was detected in concentrated culture supernatants of strain M2 and the complemented *tssB* mutant, but not in the *tssB* and *hcp* mutants. Attempts to detect Hcp secretion in the complemented *hcp* mutant were unsuccessful, indicating that this mutation may be polar on down-stream genes in the T6SS gene cluster. The presence of Hcp in culture supernatants and the reliance of Hcp secretion on a homolog of a core T6SS component (TssB) indicated that the strain M2 T6SS was functional and active under the conditions described above.

### *Acinetobacter baumannii* Out-competed *E. coli* in a T6SS-dependent Manner

**Figure 3:**
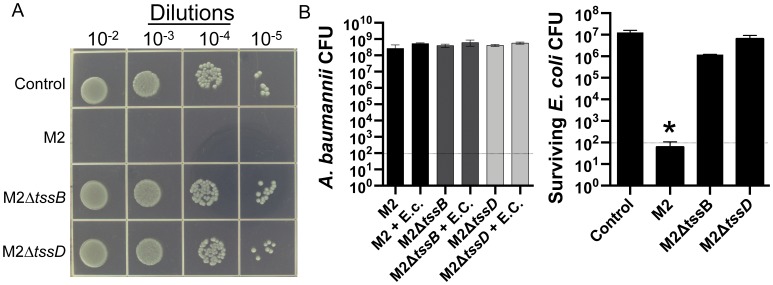
*Acinetobacter baumannii* required the products of *tssB* and *hcp* to out-compete *E. coli.* A. Semi-quantitative assessment of surviving *E. coli* after 4 h mixed with media (Control) or at a 10∶1 ratio (10 *A. baumannii* to 1 *E. coli*) with *A. baumannii* strain M2, M2Δ*tssB* or M2Δ*hcp.* B. Quantitative assessment of the *A. baumannii* and *E. coli* DH10B populations. Data represent of 3 biological replicates each performed in duplicate. Error bars represent standard error of the mean of the biological replicates. * Indicates a significant difference (*P*<0.0001) between *E. coli* CFU observed with or without co-incubation with strain M2. The dotted line on each bar graph of panel B indicates level of detection.

Since bacteria can use T6SS to compete with other bacteria and our results indicated that strain M2 expresses a functional T6SS, we hypothesized that strain M2 may utilize a T6SS to facilitate inter-bacterial competition. To study a potential role of *A. baumannii* T6SS in bacterial competition, we mixed *A. baumannii* strain M2 and *E. coli* DH10B at a 10∶1 ratio respectively, spotted this mixture onto L-agar and after 4 h of incubation, spots were excised and surviving *A. baumannii* and *E. coli* were assessed by spotting dilutions on L-agar plates with antibiotics and CFU determination. After competition with strain M2, a surviving *E. coli* population was not detected, while the surviving *E. coli* population from competitions with either M2Δ*tssB* or M2Δ*hcp* was equivalent to the no treatment control population (Figure 3.A). Quantitatively, a 5.3-log reduction (*P*<0.0001) in *E. coli* CFU was observed after co-incubation with strain M2 compared to the no treatment control. No difference was observed between the surviving *E. coli* populations that received no treatment or when co-incubated with M2Δ*hcp* (Figure 3.B). All M2 strains grew to similar population densities when spotted on L-agar and with or without co-incubation with *E. coli* (Figure 3.B). These results indicate that *A. baumannii* produces a functional T6SS, which plays a role in competition with bacteria.

### *Acinetobacter baumannii* T6SS-mediated Competition was Contact Dependent

**Figure 4:**
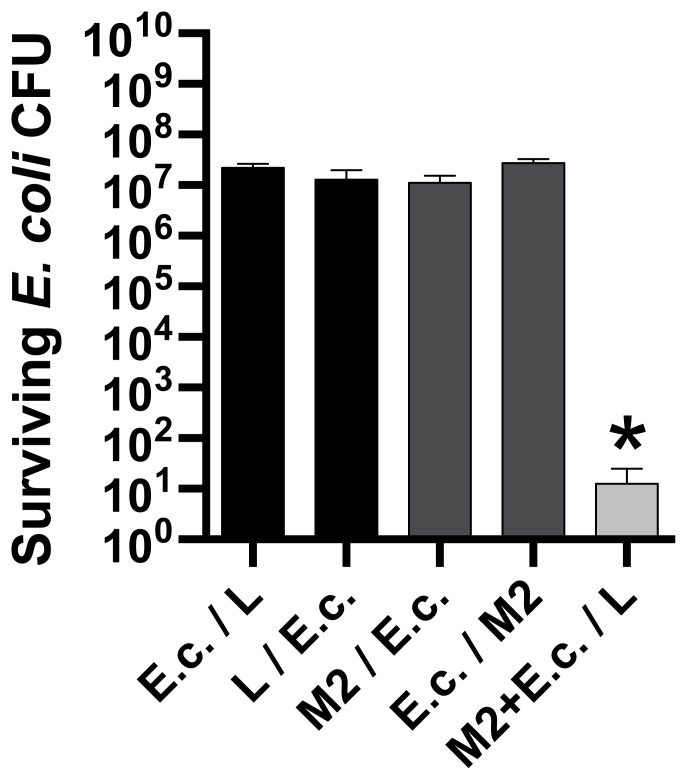
*Acinetobacter baumannii* out-competes *E. coli* in a contact dependent manner. Qualitative assessment of *E. coli* DH10B populations after 4 h of competition with the indicated *A. baumannii* strains or media. The backslash between strains (E.c. = *E. coli*, M2 = strain M2) or L (L-broth) indicate the presence of a 0.22 µm pore filter. Data represent 3 biological replicates performed in duplicate. Error bars represent standard error of the mean of the biological replicates. * Indicates a significant difference (*P*<0.01) in *E. coli* CFU between the M2+E.c./L group when compared to all other groups.

In order to further confirm that the ability of *A. baumannii* to out-compete *E. coli* was T6SS dependent, a competition experiment was performed that utilized a 0.22 µm pore filter separating strain M2 and *E. coli.* When strain M2 and *E. coli* were mixed and spotted on top of a filter (Figure 4), a ∼6-log reduction (*P*<0.01) in the *E. coli* population was observed when compared to all other groups. In addition, the *E. coli* populations incubated above or below the filter were similar, which indicated that the location of *E. coli* above or below the filter did not impact viability. When separated by the filter, no competition between strain M2 and *E. coli* was observed (Figure 4). These results provide additional evidence that *A. baumannii* uses a T6SS in inter-bacterial competition, as competition was not only dependent upon the *tssB* and *hcp* gene products, but was also contact dependent.

## Discussion

T6SS-associated genes have been identified previously in other *Acinetobacter* spp. [35] and in *A. baumannii* specifically [36], but a function for these T6SS was not elucidated. In this study, we provided the first evidence assigning a function to the *A. baumannii* T6SS in inter-bacterial competition.

Analysis of concentrated culture supernatants showed that the T6SS structural and effector protein, Hcp, accumulated in supernatants of cultures of *A. baumannii* strain M2 grown under standard laboratory conditions, and was the predominant protein species observed by Coomassie-stained SDS-PAGE of culture supernatants (Figure 2.B). In addition, Hcp secretion was dependent upon *tssB,* a gene that is predicted to encode a T6SS sheath protein that is required for T6SS function in other bacteria [50]. Western blot analysis confirmed secretion of Hcp into the culture supernatant of strain M2 and the complemented *hcp* mutant, which indicated that the *tssB* mutation did not affect expression of the downstream *hcp* gene. In addition, the *tssB* mutant produced Hcp but was unable to secrete it (Figure 2.C). Attempts to complement the Hcp secretion phenotype in the *hcp* mutant were unsuccessful. This is likely due to polar effects on down stream genes in the T6SS gene cluster. As secretion of Hcp is the hallmark of a functional T6SS [49], these results indicated that *A. baumannii* strain M2, indeed, does produce a functional T6SS. We assume, as has been postulated by others, that detection of Hcp in culture supernatants was due to Hcp being sheared off from the surface of cells that express T6SS [16], [23].

In order to identify a function for the *A. baumannii* T6SS we assessed the potential of strain M2 and the isogenic *tssB* and *hcp* mutants to outcompete *E. coli.* In this assay, strain M2 elicited a 5.3-log decrease in *E. coli* CFU during co-incubation, which was *tssB* and *hcp*-dependent (Figure 3). The CFU of *A. baumannii* strains was unaffected by co-incubation with *E. coli* (Figure 2.B). These data are similar to those reported for T6SS-dependent competition-phenotypes of *Serratia marcescens, Vibrio cholerae* and *P. aeruginosa* [21]–[23], strongly supporting the role for the *A. baumannii* T6SS in bacterial competition.

The T6SS-dependent competition between strain M2 and *E. coli* was observed, by a 6-log reduction in *E. coli* CFU when mixed and incubated together above a filter with 0.22 µm pores (Figure 4). Competition was abrogated when strain M2 and *E. coli* were separated by a filter. These results indicated that competition between *A. baumannii* and *E. coli* was dependent on cell-to-cell contact. In other systems, the antibacterial action mediated by T6SS has also been shown to be contact dependent [29]. These data provide further evidence that *A. baumannii* competition with *E. coli* was, indeed, mediated by a T6SS.

A small number of T6SS effectors, exhibiting toxicity against bacteria have been identified. In *Pseudomonas aeruginosa,* for example, the toxic effectors, Tse1, Tse2 and Tse3 are injected into target cells via a T6SS [21]. Tse1 and Tse3 are active in the periplasm and have been shown to cleave peptidoglycan. Cells expressing the cognate immunity proteins to Tse1 and Tse3, Tsi1 and Tsi2, are protected from these effects. Tse2 appears to cause quiescence when expressed in the cytoplasm in the absence the Tse2 immunity protein, Tsi2 [24]. In *S. marcescens,* the T6SS effectors Ssp1 and Ssp2 are toxic to target bacteria when localized to the periplasm in the absence of the immunity proteins Rap1a and Rap2a respectively. The mechanism behind the effect Ssp1 and Ssp2 has on target bacteria has yet to be elucidated [51]. We and others have attempted to identify potential T6SS effectors in *A. baumannii* strains using bioinformatics approaches. Russell *et al.,* using a heuristic method, identified a potential effector [25]. This predicted T6SS effector was found in one *A. baumannii* strain, strain SDF, encoded by a gene carried on a plasmid that is not conserved amongst *Acinetobacter* spp. Thus, the specific mechanism behind T6SS-dependent inter-bacterial competition in *A. baumannii* remains unclear.

The ability to compete with other bacteria, via T6SS, could be important both *in* and *ex vivo* for the success of *A. baumannii* as a pathogen. One can imagine a ventilation tube in an intubated patient becoming colonized with a variety of bacteria including *A. baumannii*. In order to colonize this surface, thrive and eventually cause disease in the patient, it is logical to assume these bacteria would compete for this niche. Similarly, in patients with infected wounds or that are afflicted with pneumonia; a mixed population of bacteria at these sites would compete to maintain the species. We postulate that *A. baumannii* utilizes its T6SS to compete with bacteria in these environments. With respect to the role of T6SS in disease, specifically T6SS-mediated interactions with eukaryotic cells, we welcome the idea that the *A. baumannii* T6SS plays a direct role in virulence but, as of yet, do not have evidence for such a role.

In conclusion, we have provided evidence that the *A. baumannii* T6SS is functional and that it plays a role in competition with other bacterial species. The specific mechanism behind this competition has yet to be elucidated. Additional studies are needed to determine if the T6SS produced by *A. baumannii* confers a fitness advantage, when faced with bacterial competitors during the course of colonization and disease. The study of biogenesis, function and regulation of *A. baumannii* T6SS, although in its infancy, has the potential to make a significant impact on our understanding of how *A. baumannii* may persist amongst mixed populations during survival in the medical environment or during the course of disease.

During revision of this manuscript, Weber *et al.* described the type VI secretion system in A. baumannii ATCC 17978 [52]. Although the gene clusters characterized by both laboratories are very similar, Weber *et al.* did not observe Type VI secretion system-dependent killing of *E. coli*. Additional work will be required to identify the basis for the differences observed in the two studies.

## Supporting Information

Table S1 Primers.
(DOCX)

Text S1 Supporting Methods.
(DOCX)
