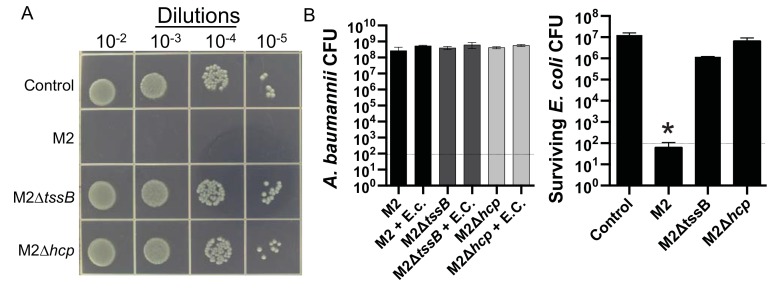# Correction: *Acinetobacter baumannii* Utilizes a Type VI Secretion System for Bacterial Competition

**DOI:** 10.1371/annotation/7aa1688c-56c8-46ca-82ea-f86697f3c4fe

**Published:** 2013-12-13

**Authors:** Michael D. Carruthers, Paul A. Nicholson, Erin N. Tracy, Robert S. Munson

Figures 1-3 are incorrect. The correct versions of Figures 1-3 can be viewed here: 

**Figure:**
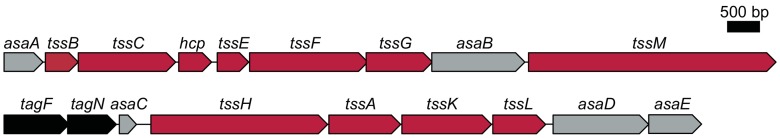


**Figure:**
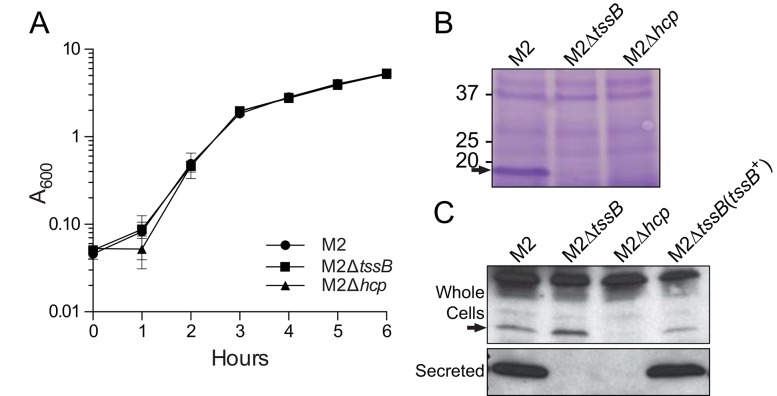


and 

**Figure:**