# Supplementary material for: Acinetobacter baumannii Utilizes a Type VI Secretion System for Bacterial Competition
Source: PLoS One. 2013 Mar 19;8(3):e59388. doi: 10.1371/journal.pone.0059388 (PMC3602014; doi:10.1371/journal.pone.0059388)
Supplement: Table S1 — Primers. (DOCX) [file pone.0059388.s001.docx]

| Table S1. Primers | | |
| --- | --- | --- |
| **Primer Set** | | **Sequence** |
| 1 | F | GCG GCA TAA CCT GAA TCT GAG |
|  | R | GAT CGA TCC TTT TTA ACC CAT CAC |
| 2 | F | GCG GAA TTC CAG CCC TTG CGC CCT GAG TG |
|  | R | GCG GAA TTC GCG AGC TCG CAT CGC CTT CTA TCG CCT TCT TG |
| 3 | F | AAG GTC AGC TAA AGA ATA ACT TTA AAT TAA AAA TAG GAA AGT TCT AAA TGA TTC CGG GGA TCC GTC GAC C |
|  | R | CAG CCT CCA CCA AAT ATT CAC TTA AAT AAT TAA GCT GCG TAA GAA GCT GTT GTA GGC TGG AGC TGC TTC |
| 4 | F | CTC CTC GAA CGA CAA CTA AAC ATA CAG T |
|  | R | CGG TAA AAG CTC TTC TAT CAC AAC ACA C |
|  | R | GCG CGG TAC CTT AAG CTG CGT AAG AAG CTG TAT T |
| 5 | F | TTT TAA TTG GTA ACT ATG TTG GTT TAT CTT G |
|  | R | GGT TTT TGA GTT GAT TGC GCA C |
| 6 | F | AAG TTC GCC TTT TGA CCC TTT AAC TAA ATA ATC TGG GAT AGA ACC TTA TGG GTC GAC GGA TCC CCG GAA T |
|  | R | CAA CAA GTG GCA TCG CTG CTG ATT GAG TAT TAT TCA TGG TCA GCC TCC GCT GTA GGC TGG AGC TGC TTC G |
| 7 | F | CGC GCG CGG CGG CCG CTG CAG CGC CCA AAC ATA ACA G |
|  | R | GCG CGC GCG CGG CCG CCG GGC CTC TTC GCT ATT ACG |
| 8 | F | CGT TGT GAC AAT TTA CCG AAC AAC TCC GCG GCC GGG AAG CCG ATC TCG GCC TCC TTA CGC ATC TGT GCG G |
|  | R | CCC TTT GTC AAC AGC AAT GGA TCG AAT TGA CAT AAG CCT GTT CGG TTC GTA GGA TCT TCA CCT AGA TCC T |
| 9 | F | CGC TAC ACC CAG TTC CCA TCT A |
|  | R | CCT AAC TTG TTC AGC AGC ATT CA |
