# Supplementary material for: Acinetobacter baumannii Utilizes a Type VI Secretion System for Bacterial Competition
Source: PLoS One. 2013 Mar 19;8(3):e59388. doi: 10.1371/journal.pone.0059388 (PMC3602014; doi:10.1371/journal.pone.0059388)
Supplement: Text S1 — Supporting Methods. (DOCX) [file pone.0059388.s002.docx]

# Text S1: Supporting Methods

**Construction of pRSM3542.** The Wanner method requires a plasmid containing a selectable marker flanked by FRT sites, which is used as a template for PCR. We constructed a template plasmid suitable for use in *A. baumannii*, designated pRSM3542, which is a derivative of Wanner’s template plasmid pKD13 [[1](#_ENREF_1)]. Initially, we cloned the Pharmacia Biotech Tn*903* kanamycin-resistance cassette into the EcoRI site of pBR322 to form pRSM574. The *sacB* gene was then amplified from pFLP2 using primer set 1 and the amplicon was ligated into pRSM574 that had been digested with StuI. The ligation product was transformed into *E. coli* DH5α, and kanamycin resistant clones were selected. A plasmid containing the *sacB* gene was identified by restriction digest, confirmed by sequencing and saved as pRSM2724. Plasmid pRSM2724 was digested with EcoRI and the resulting fragment carrying the kanamycin resistance gene and the *sacB* gene was ligated to an amplicon with EcoR1 ends generated using pKD13 as template and primers set 2. This ligation mixture was electroporated into *E. coli* EC100D, a *pir*^+^ strain and kanamycin-resistant clones were selected. A plasmid containing the kanamycin resistance gene and the *sacB* gene flanked by FRT sites was saved as pRSM3542.

**Construction of pRSM3510**

Complementation was performed using a mini-Tn*7* transposon system, which allowed insertion of genes in single copy in the strain M2 genome downstream of *glmS2*. A suicide vector that carries mini-Tn*7*-Ap, was constructed as follows. Briefly, the mini-Tn*7* cassette was amplified from pUC18T-Tn*7*-Gm [[2](#_ENREF_2)] using primer set 7. The amplicon was digested with NotI, ligated into pSMART LC-Kan (Lucigen, Middleton, WI), that had been digested with NotI, then the ligation mix was transformed into *E. coli* DH5α. Transformants were selected on L-agar containing kanamycin (20 µg/ml) and gentamicin 10 µg/ml. A plasmid from a kanamycin- and gentamicin-resistant clone was verified by sequencing and saved as pRSM3508. The *aacC1* gene in pRSM3508 was replaced with *bla* from pGEM-T-Ez (Promega) as follows. Primer set 8 was used together with pGEM-T-Ez as template to amplify a product that contained *bla* from pGEM-T-Ez flanked by 50 bp of homology to a region upstream and downstream of *aacC1* in pRSM3508. The amplicon and pRSM3508 were electroporated into *E. coli* DY380. After heat shock, as described above to induce the λ recombination system, clones were identified by plating on L-agar with ampicillin (100 µg/ml) and kanamycin (20 µg/ml). Plasmid from a single clone was verified by restriction digestion and sequencing, then saved as pRSM3509. The plasmid pRSM3509 contains a mini-Tn*7*-Ap transposon with a multiple cloning site for introduction of genes back into the *Acinetobacter* genome.

pKNOCK-Gm [[3](#_ENREF_3)] is a suicide vector that can propagate in *E. coli* strains that express the *pir* gene product but not in *A. baumannii*. We introduced the mini-Tn*7*-Ap cassette into a pKNOCK-Gm derivative expressing a kanamycin resistance gene. This plasmid contains a multiple cloning site that is similar to the multiple cloning site in the mini-Tn*7*-Ap cassette. Thus, the multiple cloning site in the plasmid backbone of pKNOCK-Km was removed by digestion with XbaI and KpnI. The ends of this digestion product were polished using the End-It DNA Repair Kit (Epicentre) and the linearized plasmid was self-ligated and transformed into *E. coli* EC100D by electroporation. A single clone was verified by restriction digest and saved as pRSM3506. The mini-Tn*7*-Ap cassette was introduced into pRSM3506 as follows. An amplicon that contained mini-Tn*7-bla*, generated by PCR with primer set 7 and pRSM3509, was digested with NotI and ligated into pRSM3506 that had been digested with NotI. Ligation mixtures were transformed into *E. coli* EC100D by electroporation and plated on L-agar containing ampicillin (50 µg/ml) and kanamycin (20 µg/ml). Plasmid from a single colony was verified by restriction digest and sequencing, then saved as pRSM3510.

**References**

1. Datsenko KA, Wanner BL (2000) One-step inactivation of chromosomal genes in *Escherichia coli* K-12 using PCR products. Proc Natl Acad Sci U S A 97: 6640-6645.

2. Choi KH, Gaynor JB, White KG, Lopez C, Bosio CM, et al. (2005) A Tn*7*-based broad-range bacterial cloning and expression system. Nat Methods 2: 443-448.

3. Alexeyev MF (1999) The pKNOCK series of broad-host-range mobilizable suicide vectors for gene knockout and targeted DNA insertion into the chromosome of gram-negative bacteria. Biotechniques 26: 824-826, 828.
